# Supplementary material for: Comparison of Bayesian methods for incorporating adult clinical trial data to improve certainty of treatment effect estimates in children
Source: PLoS One. 2023 Jun 15;18(6):e0281791. doi: 10.1371/journal.pone.0281791 (PMC10270354; doi:10.1371/journal.pone.0281791)
Supplement: S1 Table — (DOCX) [file pone.0281791.s001.docx]

**Supporting information S1 Table:** Study characteristics and event data for the outcome, no episode of vomiting in from the point of chemotherapy administration to five days afterward.

| Study author and year | Population | Age range (years) | Treatment | N participants | N events | Comparator | N participants | N events | Relative effect as reported in publication |
| --- | --- | --- | --- | --- | --- | --- | --- | --- | --- |
| Saito et al. 2013 | Adult | 25 to 86 | Fosaprepitant granisetron + dexamethasone | 173 | 62 | Granisetron + dexamethasone | 167 | 88 |  |
| Hu et al. 2013 | Adult | 20 to 77 | Aprepitant + granisetron + dexamethasone | 209 | 68 | Granisetron + dexamethasone | 212 | 93 |  |
| Rapoport et al. 2010 | Adult | NR | Aprepitant + ondansetron +dexamethasone | 430 | 138 | Ondansetron +dexamethasone | 418 | 189 |  |
| Weinstein et al. 2016 | Adult | NR | Fosaprepitant + ondansetron + dexamethasone | 502 | 115 | Ondansetron + dexamethasone | 498 | 165 |  |
| Tanioka et al. 2013 | Adult | 33 to 69 | Aprepitant + granisetron + dexamethasone | 47 | 19 | Granisetron + dexamethasone | 47 | 23 |  |
| Yahata et al. 2016 | Adult | 24 to 79 | Aprepitant + granisetron or ondansetron + dexamethasone | 155 | 64 | Granisetron or ondansetron + dexamethasone | 152 | 83 |  |
| Ito et al. 2014 | Adult | 34 to 84 | Aprepitant + 5-HT₃ receptor antagonist (type NR) + dexamethasone | 67 | 14 | 5-HT₃ receptor antagonist (Type NR) + dexamethasone | 67 | 22 |  |
| Nishimura et al. 2015 | Adult | NR | Aprepitant or Fosaprepitant + 5-HT₃ receptor antagonist (type NR) + dexamethasone | 207 | 48 | 5-HT₃ receptor antagonist (Type NR) + dexamethasone | 206 | 70 |  |
| Albany et al. 2012 | Adult | 16 to 62 | Aprepitant + 5-HT₃ receptor antagonist (except for palonosetron) + dexamethasone | 35 | 10 | 5-HT₃ receptor antagonist (except for palonosetron) + dexamethasone | 34 | 26 |  |
| Stiff et al. 2013 | Adult | 19 to 79 | Aprepitant + ondansetron +dexamethasone | 92 | 18 | Ondansetron + dexamethasone | 89 | 30 |  |
| Schmitt et al. 2014 | Adult | 27 to 71 | Aprepitant + granisetron + dexamethasone | 182 | 77 | Granisetron + dexamethasone | 181 | 107 |  |
| Hesketh et al. 2010 | Adult | NR | Aprepitant + ondansetron +dexamethasone | 520 | 165 | Ondansetron + dexamethasone | 523 | 274 |  |
| Bakhshi et al. 2015 | Children | 5 to 18 | Aprepitant + ondansetron +dexamethasone | 52 | 41 | Ondansetron + dexamethasone | 44 | 40 |  |
| Gore et al. 2009 | Children | 12 to 19 | Aprepitant + ondansetron +dexamethasone | 28 | 20 | Ondansetron + dexamethasone | 18 | 17 |  |
| Kang et al. 2015 | Children | 0 to 17 | Aprepitant + ondansetron +dexamethasone | 155 | 94 | Ondansetron + dexamethasone | 152 | 122 |  |
| Radhakrishnan et al. 2018 | Children | 1 to 12 | Fosaprepitant + ondansetron + dexamethasone | 82 | 25 | Ondansetron + dexamethasone | 82 | 48 |  |
| N.B Fosaprepitant is the intravenous version of aprepitant, which is given orally. NR= not reported in publication | | | | | | | | |  |

**S1_References**

1. Saito H, Yoshizawa H, Yoshimori K, Katakami N, Katsumata N, Kawahara M, Eguchi K. Efficacy and safety of single-dose fosaprepitant in the prevention of chemotherapy-induced nausea and vomiting in patients receiving high-dose cisplatin: a multicentre, randomised, double-blind, placebo-controlled phase 3 trial. Ann Oncol. 2013 Apr;24(4):1067-73.
2. Hu Z, Cheng Y, Zhang H, Zhou C, Han B, Zhang Y, Huang C, Chang J, Song X, Liang J, Liang H, Bai C, Yu S, Chen J, Wang J, Pan H, Chitkara DK, Hille DA, Zhang L. Aprepitant triple therapy for the prevention of chemotherapy-induced nausea and vomiting following high-dose cisplatin in Chinese patients: a randomized, double-blind, placebo-controlled phase III trial. Support Care Cancer. 2014 Apr;22(4):979-87.
3. Rapoport BL, Jordan K, Boice JA, Taylor A, Brown C, Hardwick JS, Carides A, Webb T, Schmoll HJ. Aprepitant for the prevention of chemotherapy-induced nausea and vomiting associated with a broad range of moderately emetogenic chemotherapies and tumor types: a randomized, double-blind study. Support Care Cancer. 2010 Apr;18(4):423-31.
4. Weinstein C, Jordan K, Green SA, Camacho E, Khanani S, Beckford-Brathwaite E, Vallejos W, Liang LW, Noga SJ, Rapoport BL. Single-dose fosaprepitant for the prevention of chemotherapy-induced nausea and vomiting associated with moderately emetogenic chemotherapy: results of a randomized, double-blind phase III trial. Ann Oncol. 2016 Jan;27(1):172-8.
5. Tanioka M, Kitao A, Matsumoto K, Shibata N, Yamaguchi S, Fujiwara K, Minami H, Katakami N, Morita S, Negoro S. A randomised, placebo-controlled, double-blind study of aprepitant in nondrinking women younger than 70 years receiving moderately emetogenic chemotherapy. Br J Cancer. 2013 Aug 20;109(4):859-65.
6. Yahata H, Kobayashi H, Sonoda K, Shimokawa M, Ohgami T, Saito T, Ogawa S, Sakai K, Ichinoe A, Ueoka Y, Hasuo Y, Nishida M, Masuda S, Kato K. Efficacy of aprepitant for the prevention of chemotherapy-induced nausea and vomiting with a moderately emetogenic chemotherapy regimen: a multicenter, placebo-controlled, double-blind, randomized study in patients with gynecologic cancer receiving paclitaxel and carboplatin. Int J Clin Oncol. 2016 Jun;21(3):491-7.
7. Ito Y, Karayama M, Inui N, Kuroishi S, Nakano H, Nakamura Y, Yokomura K, Toyoshima M, Shirai T, Masuda M, Yamada T, Yasuda K, Hayakawa H, Suda T, Chida K. Aprepitant in patients with advanced non-small-cell lung cancer receiving carboplatin-based chemotherapy. Lung Cancer. 2014 Jun;84(3):259-64.
8. Nishimura J, Satoh T, Fukunaga M, Takemoto H, Nakata K, Ide Y, Fukuzaki T, Kudo T, Miyake Y, Yasui M, Morita S, Sakai D, Uemura M, Hata T, Takemasa I, Mizushima T, Ohno Y, Yamamoto H, Sekimoto M, Nezu R, Doki Y, Mori M; Multi-center Clinical Study Group of Osaka, Colorectal Cancer Treatment Group (MCSGO). Combination antiemetic therapy with aprepitant/fosaprepitant in patients with colorectal cancer receiving oxaliplatin-based chemotherapy (SENRI trial): a multicentre, randomised, controlled phase 3 trial. Eur J Cancer. 2015 Jul;51(10):1274-82.
9. Albany C, Brames MJ, Fausel C, Johnson CS, Picus J, Einhorn LH. Randomized, double-blind, placebo-controlled, phase III cross-over study evaluating the oral neurokinin-1 antagonist aprepitant in combination with a 5HT3 receptor antagonist and dexamethasone in patients with germ cell tumors receiving 5-day cisplatin combination chemotherapy regimens: a hoosier oncology group study. J Clin Oncol. 2012 Nov 10;30(32):3998-4003.
10. Stiff PJ, Fox-Geiman MP, Kiley K, Rychlik K, Parthasarathy M, Fletcher-Gonzalez D, Porter N, Go A, Smith SE, Rodriguez TE. Prevention of nausea and vomiting associated with stem cell transplant: results of a prospective, randomized trial of aprepitant used with highly emetogenic preparative regimens. Biol Blood Marrow Transplant. 2013 Jan;19(1):49-55.e1
11. Schmitt T, Goldschmidt H, Neben K, Freiberger A, Hüsing J, Gronkowski M, Thalheimer M, Pelzl le H, Mikus G, Burhenne J, Ho AD, Egerer G. Aprepitant, granisetron, and dexamethasone for prevention of chemotherapy-induced nausea and vomiting after high-dose melphalan in autologous transplantation for multiple myeloma: results of a randomized, placebo-controlled phase III trial. J Clin Oncol. 2014 Oct 20;32(30):3413-20.
12. Hesketh PJ, Aapro M, Street JC, Carides AD. Evaluation of risk factors predictive of nausea and vomiting with current standard-of-care antiemetic treatment: analysis of two phase III trials of aprepitant in patients receiving cisplatin-based chemotherapy. Support Care Cancer. 2010 Sep;18(9):1171-7.
13. Bakhshi S, Batra A, Biswas B, Dhawan D, Paul R, Sreenivas V. Aprepitant as an add-on therapy in children receiving highly emetogenic chemotherapy: a randomized, double-blind, placebo-controlled trial. Support Care Cancer. 2015 Nov;23(11):3229-37.
14. Gore L, Chawla S, Petrilli A, Hemenway M, Schissel D, Chua V, Carides AD, Taylor A, Devandry S, Valentine J, Evans JK, Oxenius B; Adolescent Aprepitant in Cancer Study Group. Aprepitant in adolescent patients for prevention of chemotherapy-induced nausea and vomiting: a randomized, double-blind, placebo-controlled study of efficacy and tolerability. Pediatr Blood Cancer. 2009 Feb;52(2):242-7.
15. Kang HJ, Loftus S, Taylor A, DiCristina C, Green S, Zwaan CM. Aprepitant for the prevention of chemotherapy-induced nausea and vomiting in children: a randomised, double-blind, phase 3 trial. Lancet Oncol. 2015 Apr;16(4):385-94.
16. Radhakrishnan V, Joshi A, Ramamoorthy J, Rajaraman S, Ganesan P, Ganesan TS, Dhanushkodi M, Sagar TG. Intravenous fosaprepitant for the prevention of chemotherapy-induced vomiting in children: A double-blind, placebo-controlled, phase III randomized trial. Pediatr Blood Cancer. 2019 Mar;66(3):e27551.
