# Supplementary material for: Comparison of Bayesian methods for incorporating adult clinical trial data to improve certainty of treatment effect estimates in children
Source: PLoS One. 2023 Jun 15;18(6):e0281791. doi: 10.1371/journal.pone.0281791 (PMC10270354; doi:10.1371/journal.pone.0281791)
Supplement: S2 Table — (DOCX) [file pone.0281791.s002.docx]

**Supporting information S3 Table:** Results from MA and the proportional effects ‘cut function’ model comparing aprepitant or fosaprepitant to control regimen for the treatment of chemotherapy induced nausea and vomiting.

| **Model** | **Number of data points** | **Fixed effect model** | | | **Random effect model** | | | |
| --- | --- | --- | --- | --- | --- | --- | --- | --- |
|  |  | **RR (95%CrI)** | **DIC** | **totresdev*** | **RR (95%CrI)** | **Between-study heterogeneity (SD)** | **DIC** | **totresdev*** |
| Adults’ data only | 24 | 0.672 (0.626 to 0.720) | 165.7 | 23.72 | 0.672 (0.614 to 0.731) | 0.064(0.005 to 0.189) | 167.2 | 22.88 |
| Children’s data only | 8 | 0.768 (0.689 to 0.849) | 48.6 | 11.32 | 0.754 (0.468 to 1.1) | 0.287 (0.01 to 1.140) | 48.1 | 8.54 |
| **Proportional effects model using ‘cut function’** | | | | | | | | |
| Adults | 32 | 0.672 (0.626 to 0.721) | 233.7 | 37.83 | 0.671 (0.618 to 0.731) | 0.057 (0.003 to 0.160) | 232.9 | 34.83 |
| Children |  | 0.767 (0.671 to 0.868) |  |  | 0.759 (0.659 to 0.861) |  |  |  |
| Lambda (relative risk scale) |  | 1.142 (1.009 to 1.283) | | | 1.132 (0.960 to 1.311) | | | |
