# Supplementary material for: Comparison of Bayesian methods for incorporating adult clinical trial data to improve certainty of treatment effect estimates in children
Source: PLoS One. 2023 Jun 15;18(6):e0281791. doi: 10.1371/journal.pone.0281791 (PMC10270354; doi:10.1371/journal.pone.0281791)
Supplement: S1 File — (DOCX) [file pone.0281791.s003.docx]

**Supporting information S2 Open BUGS code:** Code for implementing the information sharing models described in this paper.

Models can be implemented in the statistical software programme WinBUGS or OpenBUGS.

The ‘lumping’ model

Depending on the data included, this code will run an NMA separately for adults and children and 'combined' if both data sets are included, where we assume there is no difference in treatment effect between the two populations.

Fixed effects model (relative risk)

# Binomial likelihood, log-Relative Risk

# Fixed effects model

model{ # *** PROGRAM STARTS

for(i in 1:ns){ # LOOP THROUGH STUDIES

mu[i] <- log(p[i,1])

p[i,1] ~ dunif(0,1) # vague priors for all trial baselines

for (k in 1:na[i]){ # LOOP THROUGH ARMS

r[i,k] ~ dbin(p[i,k],n[i,k]) # binomial likelihood

rhat[i,k] <- p[i,k] * n[i,k] # expected value of the numerators

# Deviance contribution

dev[i,k] <- 2 * (r[i,k] * (log(r[i,k])-log(rhat[i,k]))

+ (n[i,k]-r[i,k]) * (log(n[i,k]-r[i,k]) - log(n[i,k]-rhat[i,k])))

}

# model for linear predictor

for (k in 2:na[i]){

log(p[i,k]) <- mu[i] + min(delta[i,k], -log(p[i,1]))

delta[i,k] <- d[t[i,k]] - d[t[i,1]] # fixed effect model

}

# summed residual deviance contribution for this trial

resdev[i] <- sum(dev[i,1:na[i]])

}

totresdev <- sum(resdev[]) # Total Residual Deviance

#

d[1]<-0 # treatment effect is zero for reference treatment

# vague priors for treatment effects

for (k in 2:nt){ d[k] ~ dnorm(0,.0001) }

# pairwise RRs for all possible pair-wise comparisons

for (c in 1:(nt-1)){

for (k in (c+1):nt){

lnRR[c,k] <- d[k] - d[c]

RR[c,k] <- exp(d[k] - d[c])

}

}

# ranking on relative scale

for (k in 1:nt) {

# rk[k] <- nt+1-rank(d[],k) # assumes events are "good"

rk[k] <- rank(d[],k) # assumes events are "bad"

best[k] <- equals(rk[k],1) # calculate probability that treat k is best

# calculates probability that treat k is h-th best

for (h in 1:nt){ prob[h,k] <- equals(rk[k],h) }

}

} # *** PROGRAM ENDS

Random effects model (relative risk)

# Binomial likelihood, log-Relative Risk

# Random effects model for multi-arm trials

model{ # *** PROGRAM STARTS

for(i in 1:ns){ # LOOP THROUGH STUDIES

w[i,1] <- 0 # adjustment for multi-arm trials is zero for control arm

delta[i,1] <- 0 # treatment effect is zero for control arm

**mu[i] <- log(p[i,1])**

**p[i,1] ~ dunif(0,1)** # vague priors for all trial baselines

for (k in 1:na[i]) { # LOOP THROUGH ARMS

r[i,k] ~ dbin(p[i,k],n[i,k]) # binomial likelihood

rhat[i,k] <- p[i,k] * n[i,k] # expected value of the numerators

# Deviance contribution

dev[i,k] <- 2 * (r[i,k] * (log(r[i,k])-log(rhat[i,k]))

+ (n[i,k]-r[i,k]) * (log(n[i,k]-r[i,k]) - log(n[i,k]-rhat[i,k])))

}

# summed residual deviance contribution for this trial

resdev[i] <- sum(dev[i,1:na[i]])

for (k in 2:na[i]) { # LOOP THROUGH ARMS

**log(p[i,k]) <- mu[i] + min(delta[i,k], -log(p[i,1]))**

# trial-specific LRR distributions

delta[i,k] ~ dnorm(md[i,k],taud[i,k])

# mean of LRR distributions (with multi-arm trial correction)

md[i,k] <- d[t[i,k]] - d[t[i,1]] + sw[i,k]

# precision of LRR distributions (with multi-arm trial correction)

taud[i,k] <- tau *2*(k-1)/k

# adjustment for multi-arm RCTs

w[i,k] <- (delta[i,k] - d[t[i,k]] + d[t[i,1]])

# cumulative adjustment for multi-arm trials

sw[i,k] <- sum(w[i,1:k-1])/(k-1)

}

}

totresdev <- sum(resdev[]) # Total Residual Deviance

d[1] <- 0 # treatment effect is zero for reference treatment

# vague priors for treatment effects

for (k in 2:nt){ d[k] ~ **dnorm(0,.0001)** }

sd ~ dunif(0,2) # vague prior for between-trial SD

tau <- pow(sd,-2) # between-trial precision = (1/between-trial variance)

# pairwise RRs and LRRs for all possible pair-wise comparisons

for (c in 1:(nt-1)) {

for (k in (c+1):nt) {

**lnRR[c,k] <- d[k] - d[c]**

**RR[c,k] <- exp(d[k] - d[c])**

}

}

# ranking on relative scale

for (k in 1:nt) {

# rk[k] <- nt+1-rank(d[],k) # assumes events are "good"

rk[k] <- rank(d[],k) # assumes events are "bad"

best[k] <- equals(rk[k],1) # calculate probability that treat k is best

# calculates probability that treat k is h-th best

for (h in 1:nt){ prob[h,k] <- equals(rk[k],h) }

}

} # *** PROGRAM ENDS

END

Example of data structure:

list(ns=4, nt=2 )

t[,1] r[,1] n[,1] t[,2] r[,2] n[,2] na[] # study

1 40 44 2 41 52 2 # 1

1 17 18 2 20 28 2 # 2

Initial values

#FE chains

list(d=c(NA,0))

list(d=c(NA,1))

list(d=c(NA,2))

#RE chains

list(d=c(NA,0), sd=0.1)

list(d=c(NA,1),sd=0.5)

list(d=c(NA,1),sd=0.3)

The ‘splitting’ model

Fixed effects model (relative risk)

# Binomial likelihood, log-Relative Risk

# Fixed effects model - 2 populations

model{ # *** PROGRAM STARTS

for(i in 1:ns){ # LOOP THROUGH ALL STUDIES

# The priors for trial baseline are defined for each study

mu[i] <- log(p[i,1])

p[i,1] ~ dunif(0,1) # vague priors for all trial baselines

# The likelihood, expected value of numerators and deviance for each arm of each study

for (k in 1:na[i]){ # LOOP THROUGH ARMS

r[i,k] ~ dbin(p[i,k],n[i,k]) # binomial likelihood

rhat[i,k] <- p[i,k] * n[i,k] # expected value of the numerators

# Deviance contribution

dev[i,k] <- 2 * (r[i,k] * (log(r[i,k])-log(rhat[i,k]))

+ (n[i,k]-r[i,k]) * (log(n[i,k]-r[i,k]) - log(n[i,k]-rhat[i,k])))

} # close loop for the arms

# The residual deviance for each study

# summed residual deviance contribution for each trial

resdev[i] <- sum(dev[i,1:na[i]])

} # close loop for the studies

# models for linear predictors-

# This has been moved out the 'study' loop because you want to estimate the d's separately for adults and children.

#

**# ADULTS**

for(i in 1:nsA){

for (k in 2:na[i]){

log(p[i,k]) <- mu[i] + min(delta[i,k], -log(p[i,1]))

delta[i,k] <- d[1,t[i,k]] - d[1,t[i,1]] # this part editied for 1=adults and 2=children

}

}

**# CHILDREN**

for(i in (nsA+1):(nsA+nsC)){

for (k in 2:na[i]){

log(p[i,k]) <- mu[i] + min(delta[i,k], -log(p[i,1]))

delta[i,k] <- d[2,t[i,k]] - d[2,t[i,1]] # this part editied for 1=adults and 2=children

}

}

totresdev <- sum(resdev[]) # Total Residual Deviance

#

for (j in 1:2){ # LOOP OVER POPULATIONS 1=adults; 2=children

d[j,1] <- 0 # treatment effect is zero for reference treatment

# vague priors for treatment effects

for (k in 2:nt){ d[j,k] ~ dnorm(0,.0001)}

# pairwise RRs for all possible pair-wise comparisons

for (c in 1:(nt-1)){

for (k in (c+1):nt){

lnRR[j,c,k] <- d[j,k] - d[j,c]

RR[j,c,k] <- exp(d[j,k] - d[j,c])

}

}

# ranking on relative scale

for (k in 1:nt) {

# rk[j,k] <- nt+1-rank(d[j, ],k) # assumes events are "good"

rk[j,k] <- rank(d[j, ],k) # assumes events are "bad"

best[j,k] <- equals(rk[j,k],1) #calculate probability that treat k is best

# calculates probability that treat k is h-th best

for (h in 1:nt){ prob[j,h,k] <- equals(rk[j,k],h) }

}

} # close loop over populations

} # *** PROGRAM END

Random effects model (relative risk)

# Binomial likelihood, log-Relative Risk

# Random effects model for multi-arm trials - 2 populations

model{ # *** PROGRAM STARTS

for(i in 1:ns){ # LOOP THROUGH STUDIES

w[i,1] <- 0 # adjustment for multi-arm trials is zero for control arm

delta[i,1] <- 0 # treatment effect is zero for control arm

**mu[i] <- log(p[i,1])**

**p[i,1] ~ dunif(0,1)** # vague priors for all trial baselines

for (k in 1:na[i]) { # LOOP THROUGH ARMS

r[i,k] ~ dbin(p[i,k],n[i,k]) # binomial likelihood

rhat[i,k] <- p[i,k] * n[i,k] # expected value of the numerators

#Deviance contribution

dev[i,k] <- 2 * (r[i,k] * (log(r[i,k])-log(rhat[i,k]))

+ (n[i,k]-r[i,k]) * (log(n[i,k]-r[i,k]) - log(n[i,k]-rhat[i,k])))

} # close loop for the arms

# summed residual deviance contribution for this trial

resdev[i] <- sum(dev[i,1:na[i]])

} # close loop for the studies

# models for linear predictors

#

**# ADULTS**

for(i in 1:nsA){

for (k in 2:na[i]) { # LOOP THROUGH ARMS

**log(p[i,k]) <- mu[i] + min(delta[i,k], -log(p[i,1]))**

# trial-specific LRR distributions

delta[i,k] ~ dnorm(md[i,k],taud[i,k])

# mean of LRR distributions (with multi-arm trial correction)

md[i,k] <- d[1,t[i,k]] - d[1,t[i,1]] + sw[i,k]

# precision of LOR distributions (with multi-arm trial correction)

taud[i,k] <- tau *2*(k-1)/k

# adjustment for multi-arm RCTs

w[i,k] <- (delta[i,k] - d[1,t[i,k]] + d[1,t[i,1]])

# cumulative adjustment for multi-arm trials

sw[i,k] <- sum(w[i,1:k-1])/(k-1)

} # CLOSE LOOP FOR ARMS

}

**# CHILDREN**

for(i in (nsA+1):(nsA+nsC)){

for (k in 2:na[i]) { # LOOP THROUGH ARMS

**log(p[i,k]) <- mu[i] + min(delta[i,k], -log(p[i,1]))**

# trial-specific LRR distributions

delta[i,k] ~ dnorm(md[i,k],taud[i,k])

# mean of LRR distributions (with multi-arm trial correction)

md[i,k] <- d[2,t[i,k]] - d[2,t[i,1]] + sw[i,k]

# precision of LRR distributions (with multi-arm trial correction)

taud[i,k] <- tau *2*(k-1)/k

# adjustment for multi-arm RCTs

w[i,k] <- (delta[i,k] - d[2,t[i,k]] + d[2,t[i,1]])

# cumulative adjustment for multi-arm trials

sw[i,k] <- sum(w[i,1:k-1])/(k-1)

} # CLOSE LOOP FOR ARMS

}

totresdev <- sum(resdev[]) # Total Residual Deviance

sd ~ dunif(0,2) # vague prior for between-trial SD

tau <- pow(sd,-2) # between-trial precision = (1/between-trial variance)

#

for (j in 1:2){ # LOOP OVER POPULATIONS 1=adults; 2=children

d[j,1] <- 0 # treatment effect is zero for reference treatment

# vague priors for treatment effects

for (k in 2:nt){ d[j,k] ~ dnorm(0,.0001) }

# pairwise RRs for all possible pair-wise comparisons

for (c in 1:(nt-1)){

for (k in (c+1):nt){

lnRR[j,c,k] <- d[j,k] - d[j,c]

RR[j,c,k] <- exp(d[j,k] - d[j,c])

}

}

# ranking on relative scale

for (k in 1:nt) {

# rk[j,k] <- nt+1-rank(d[j, ],k) # assumes events are "good"

rk[j,k] <- rank(d[j, ],k) # assumes events are "bad"

best[j,k] <- equals(rk[j,k],1) #calculate probability that treat k is best

# calculates probability that treat k is h-th best

for (h in 1:nt){ prob[j,h,k] <- equals(rk[j,k],h) }

}

} # close loop over populations

} # *** PROGRAM ENDS

Example of data structure:

list(ns=8, nt=2, nsA=4, nsC=4) # indicating 8 studies in total, comparing 2 treatments from 4 adult studies ad 4 children’s studies

t[,1] r[,1] n[,1] t[,2] r[,2] n[,2] na[] #study

1 88 167.00 2 62 173 2 # 1

1 93 212 2 68 209 2 # 2

1 189 418 2 138 430 2 # 3

1 165 498 2 115 502 2 # 4

1 23 47 2 19 47 2 # 5

1 83 152 2 64 155 2 # 6

1 22 67 2 14 67 2 # 7

1 70 206 2 48 207 2 # 8

Initial values

**#FE chains**

#chain 1

list(d = structure(.Data = c(NA, 1, NA, NA), .Dim = c(2,2)), lambda=c(NA,0) )

#chain 2

list(d = structure(.Data = c(NA, 3, NA, NA), .Dim = c(2,2)), lambda=c(NA,-2) )

#chain 3

list(d = structure(.Data = c(NA, 2, NA, NA), .Dim = c(2,2)), lambda=c(NA,1) )

**#RE chains**

#chain 1

list(d = structure(.Data = c(NA, 1, NA, NA), .Dim = c(2,2)), sd=0.1, lambda=c(NA,0) )

#chain 2

list(d = structure(.Data = c(NA, 3, NA, NA), .Dim = c(2,2)), sd=0.3, lambda=c(NA,-2) )

#chain 3

list(d = structure(.Data = c(NA, 2, NA, NA), .Dim = c(2,2)), sd=0.5, lambda=c(NA,1) )

Proportional effects model

Fixed effects model (relative risk)

# Binomial likelihood, log-Relative Risk

# Fixed effects model - 2 populations, functional relationship 'lambda'

model{ # *** PROGRAM STARTS

for(i in 1:ns){ # LOOP THROUGH STUDIES

mu[i] <- log(p[i,1])

p[i,1] ~ dunif(0,1) # vague priors for all trial baselines

for (k in 1:na[i]){ # LOOP THROUGH ARMS

r[i,k] ~ dbin(p[i,k],n[i,k]) # binomial likelihood

rhat[i,k] <- p[i,k] * n[i,k] # expected value of the numerators

# Deviance contribution

dev[i,k] <- 2 * (r[i,k] * (log(r[i,k])-log(rhat[i,k]))

+ (n[i,k]-r[i,k]) * (log(n[i,k]-r[i,k]) - log(n[i,k]-rhat[i,k])))

} # close loop for the arms

# The residual deviance for each study

# summed residual deviance contribution for this trial

resdev[i] <- sum(dev[i,1:na[i]])

} # close loop for the studies

# models for linear predictors-

**# ADULTS**

for(i in 1:nsA){

for (k in 2:na[i]){

log(p[i,k]) <- mu[i] + min(delta[i,k], -log(p[i,1]))

delta[i,k] <- d[1,t[i,k]] - d[1,t[i,1]] # - this part edited for adults and children

}

}

**# CHILDREN**

for(i in (nsA+1):(nsA+nsC)){

for (k in 2:na[i]){

log(p[i,k]) <- mu[i] + min(delta[i,k], -log(p[i,1]))

delta[i,k] <- d[2,t[i,k]] - d[2,t[i,1]] # - this part edited for adults and children

}

}

totresdev <- sum(resdev[]) # Total Residual Deviance

for (k in 2:nt){

d[1,k] ~ dnorm(0,.0001) # vague prior for adult treat effect

d[2,k] <- d[1,k] + lambda[k]  **# FUNCTIONAL RELATIONSHIP for children**

lambda[k] ~ dnorm(0,0.001) # non-inf prior for sharing parameter

explambda[k] <- exp(lambda[k])

}

for (j in 1:2){ # LOOP OVER POPULATIONS 1=adults; 2=children

d[j,1] <- 0 # treatment effect is zero for reference treatment

# pairwise RRs for all possible pair-wise comparisons

for (c in 1:(nt-1)){

for (k in (c+1):nt){

lnRR[j,c,k] <- d[j,k] - d[j,c]

RR[j,c,k] <- exp(d[j,k] - d[j,c])

}

}

# ranking on relative scale

for (k in 1:nt) {

# rk[j,k] <- nt+1-rank(d[j, ],k) # assumes events are "good"

rk[j,k] <- rank(d[j, ],k) # assumes events are "bad"

best[j,k] <- equals(rk[j,k],1) #calculate probability that treat k is best

# calculates probability that treat k is h-th best

for (h in 1:nt){ prob[j,h,k] <- equals(rk[j,k],h) }

}

} # close loop over populations

} # *** PROGRAM ENDS

Random effects model (relative risk)

# Binomial likelihood, log-Relative Risk

# Random effects model for multi-arm trials, functional relationship 'lambda'

model{ # *** PROGRAM STARTS

for(i in 1:ns){ # LOOP THROUGH STUDIES

w[i,1] <- 0 # adjustment for multi-arm trials is zero for control arm

delta[i,1] <- 0 # treatment effect is zero for control arm

**mu[i] <- log(p[i,1])**

**p[i,1] ~ dunif(0,1)** # vague priors for all trial baselines

for (k in 1:na[i]) { # LOOP THROUGH ARMS

r[i,k] ~ dbin(p[i,k],n[i,k]) # binomial likelihood

rhat[i,k] <- p[i,k] * n[i,k] # expected value of the numerators

#Deviance contribution

dev[i,k] <- 2 * (r[i,k] * (log(r[i,k])-log(rhat[i,k]))

+ (n[i,k]-r[i,k]) * (log(n[i,k]-r[i,k]) - log(n[i,k]-rhat[i,k])))

} # CLOSE LOOP FOR ARMS

# summed residual deviance contribution for this trial

resdev[i] <- sum(dev[i,1:na[i]])

} # CLOSE LOOP FOR STUDIES

# models for linear predictors

#

**# ADULTS**

for(i in 1:nsA){

for (k in 2:na[i]) { # LOOP THROUGH ARMS

**log(p[i,k]) <- mu[i] + min(delta[i,k], -log(p[i,1]))**

# trial-specific LOR distributions

delta[i,k] ~ dnorm(md[i,k],taud[i,k])

# mean of LOR distributions (with multi-arm trial correction)

md[i,k] <- d[1,t[i,k]] - d[1,t[i,1]] + sw[i,k]

# precision of LOR distributions (with multi-arm trial correction)

taud[i,k] <- tau *2*(k-1)/k

# adjustment for multi-arm RCTs

w[i,k] <- (delta[i,k] - d[1,t[i,k]] + d[1,t[i,1]])

# cumulative adjustment for multi-arm trials

sw[i,k] <- sum(w[i,1:k-1])/(k-1)

} # CLOSE LOOP FOR ARMS

}

**# CHILDREN**

for(i in (nsA+1):(nsA+nsC)){

for (k in 2:na[i]) { # LOOP THROUGH ARMS

**log(p[i,k]) <- mu[i] + min(delta[i,k], -log(p[i,1]))**

# trial-specific LOR distributions

delta[i,k] ~ dnorm(md[i,k],taud[i,k])

# mean of LOR distributions (with multi-arm trial correction)

md[i,k] <- d[2,t[i,k]] - d[2,t[i,1]] + sw[i,k]

# precision of LOR distributions (with multi-arm trial correction)

taud[i,k] <- tau *2*(k-1)/k

# adjustment for multi-arm RCTs

w[i,k] <- (delta[i,k] - d[2,t[i,k]] + d[2,t[i,1]])

# cumulative adjustment for multi-arm trials

sw[i,k] <- sum(w[i,1:k-1])/(k-1)

} # CLOSE LOOP FOR ARMS

}

totresdev <- sum(resdev[]) # Total Residual Deviance

sd ~ dunif(0,2) # vague prior for between-trial SD

tau <- pow(sd,-2) # between-trial precision = (1/between-trial variance)

for (k in 2:nt){

d[1,k] ~ dnorm(0,.0001) # vague prior for adult treat effect

d[2,k] <- d[1,k] + lambda[k]  **# FUNCTIONAL RELATIONSHIP for children**

lambda[k] ~ dnorm(0,0.001) # non-inf prior for sharing parameter

explambda[k] <- exp(lambda[k])

}

for (j in 1:2){ # LOOP OVER POPULATIONS 1=adults; 2=children

d[j,1] <- 0 # treatment effect is zero for reference treatment

# pairwise RRs for all possible pair-wise comparisons

for (c in 1:(nt-1)){

for (k in (c+1):nt){

lnRR[j,c,k] <- d[j,k] - d[j,c]

RR[j,c,k] <- exp(d[j,k] - d[j,c])

}

}

# ranking on relative scale

for (k in 1:nt) {

# rk[j,k] <- nt+1-rank(d[j, ],k) # assumes events are "good"

rk[j,k] <- rank(d[j, ],k) # assumes events are "bad"

best[j,k] <- equals(rk[j,k],1) #calculate probability that treat k is best

# calculates probability that treat k is h-th best

for (h in 1:nt){ prob[j,h,k] <- equals(rk[j,k],h) }

}

} # close loop over populations

} # *** PROGRAM ENDS

*Suggested data format and initial values are the same as for the splitting model.*

Proportional effects mode using ‘cut function’:

Fixed effects model – (Relative Risk)

# Binomial likelihood, log-Relative Risk

# Fixed effects model - 2 populations, functional relationship 'lambda' with CUT function

model{ # *** PROGRAM STARTS

for(i in 1:ns){ # LOOP THROUGH STUDIES

mu[i] <- log(p[i,1])

p[i,1] ~ dunif(0,1) # vague priors for all trial baselines

for (k in 1:na[i]){ # LOOP THROUGH ARMS

r[i,k] ~ dbin(p[i,k],n[i,k]) # binomial likelihood

rhat[i,k] <- p[i,k] * n[i,k] # expected value of the numerators

# Deviance contribution

dev[i,k] <- 2 * (r[i,k] * (log(r[i,k])-log(rhat[i,k]))

+ (n[i,k]-r[i,k]) * (log(n[i,k]-r[i,k]) - log(n[i,k]-rhat[i,k])))

} # close loop for the arms

# The residual deviance for each study

# summed residual deviance contribution for this trial

resdev[i] <- sum(dev[i,1:na[i]])

} # close loop for the studies

# models for linear predictors-

#

**# ADULTS**

for(i in 1:nsA){

for (k in 2:na[i]){

log(p[i,k]) <- mu[i] + min(delta[i,k], -log(p[i,1]))

delta[i,k] <- d[1,t[i,k]] - d[1,t[i,1]] # - this part editied for adults and children

}

}

**# CHILDREN**

for(i in (nsA+1):(nsA+nsC)){

for (k in 2:na[i]){

log(p[i,k]) <- mu[i] + min(delta[i,k], -log(p[i,1]))

delta[i,k] <- d[2,t[i,k]] - d[2,t[i,1]] # - this part editied for adults and children

}

}

totresdev <- sum(resdev[]) # Total Residual Deviance

for (k in 2:nt){

d[1,k] ~ dnorm(0,.0001) # vague prior for adult treat effect

### CUT children's effect so it does not affect adult effect ###

d[2,k] <- d.cut[1,k] + lambda[k]  **# FUNCTIONAL RELATIONSHIP for children**

d.cut[1,k] <- cut(d[1,k]) # CUT children's d

lambda[k] ~ dnorm(0,0.001) # non-inf prior for sharing parameter

explambda[k] <- exp(lambda[k])

}

for (j in 1:2){ # LOOP OVER POPULATIONS 1=adults; 2=children

d[j,1] <- 0 # treatment effect is zero for reference treatment

# pairwise RRs for all possible pair-wise comparisons

for (c in 1:(nt-1)){

for (k in (c+1):nt){

lnRR[j,c,k] <- d[j,k] - d[j,c]

RR[j,c,k] <- exp(d[j,k] - d[j,c])

}

}

# ranking on relative scale

for (k in 1:nt) {

# rk[j,k] <- nt+1-rank(d[j, ],k) # assumes events are "good"

rk[j,k] <- rank(d[j, ],k) # assumes events are "bad"

best[j,k] <- equals(rk[j,k],1) #calculate probability that treat k is best

# calculates probability that treat k is h-th best

for (h in 1:nt){ prob[j,h,k] <- equals(rk[j,k],h) }

}

} # close loop over populations

} # *** PROGRAM ENDS

Random effects model (relative risk)

# Binomial likelihood, log-Relative Risk

# Random effects model for multi-arm trials, functional relationship 'lambda'

# with CUT function

model{ # *** PROGRAM STARTS

for(i in 1:ns){ # LOOP THROUGH STUDIES

w[i,1] <- 0 # adjustment for multi-arm trials is zero for control arm

delta[i,1] <- 0 # treatment effect is zero for control arm

**mu[i] <- log(p[i,1])**

**p[i,1] ~ dunif(0,1)** # vague priors for all trial baselines

for (k in 1:na[i]) { # LOOP THROUGH ARMS

r[i,k] ~ dbin(p[i,k],n[i,k]) # binomial likelihood

rhat[i,k] <- p[i,k] * n[i,k] # expected value of the numerators

#Deviance contribution

dev[i,k] <- 2 * (r[i,k] * (log(r[i,k])-log(rhat[i,k]))

+ (n[i,k]-r[i,k]) * (log(n[i,k]-r[i,k]) - log(n[i,k]-rhat[i,k])))

} # CLOSE LOOP FOR ARMS

# summed residual deviance contribution for this trial

resdev[i] <- sum(dev[i,1:na[i]])

} # CLOSE LOOP FOR STUDIES

# models for linear predictors

#

**# ADULTS**

for(i in 1:nsA){

for (k in 2:na[i]) { # LOOP THROUGH ARMS

**log(p[i,k]) <- mu[i] + min(delta[i,k], -log(p[i,1]))**

# trial-specific LOR distributions

delta[i,k] ~ dnorm(md[i,k],taud[i,k])

# mean of LOR distributions (with multi-arm trial correction)

md[i,k] <- d[1,t[i,k]] - d[1,t[i,1]] + sw[i,k]

# precision of LOR distributions (with multi-arm trial correction)

taud[i,k] <- tau *2*(k-1)/k

# adjustment for multi-arm RCTs

w[i,k] <- (delta[i,k] - d[1,t[i,k]] + d[1,t[i,1]])

# cumulative adjustment for multi-arm trials

sw[i,k] <- sum(w[i,1:k-1])/(k-1)

} # CLOSE LOOP FOR ARMS

}

**# CHILDREN**

for(i in (nsA+1):(nsA+nsC)){

for (k in 2:na[i]) { # LOOP THROUGH ARMS

**log(p[i,k]) <- mu[i] + min(delta[i,k], -log(p[i,1]))**

# trial-specific LOR distributions

delta[i,k] ~ dnorm(md[i,k],taud[i,k])

# mean of LOR distributions (with multi-arm trial correction)

md[i,k] <- d[2,t[i,k]] - d[2,t[i,1]] + sw[i,k]

# precision of LOR distributions (with multi-arm trial correction)

taud[i,k] <- tau *2*(k-1)/k

# adjustment for multi-arm RCTs

w[i,k] <- (delta[i,k] - d[2,t[i,k]] + d[2,t[i,1]])

# cumulative adjustment for multi-arm trials

sw[i,k] <- sum(w[i,1:k-1])/(k-1)

} # CLOSE LOOP FOR ARMS

}

totresdev <- sum(resdev[]) # Total Residual Deviance

sd ~ dunif(0,2) # vague prior for between-trial SD

tau <- pow(sd,-2) # between-trial precision = (1/between-trial variance)

for (k in 2:nt){

d[1,k] ~ dnorm(0,.0001) # vague prior for adult treat effect

### CUT children's effect so it does not affect adult d ###

d[2,k] <- d.cut[1,k] + lambda[k]  **# FUNCTIONAL RELATIONSHIP for children**

d.cut[1,k] <- cut(d[1,k]) # CUT children's d

lambda[k] ~ dnorm(0,0.001) # non-inf prior for sharing parameter

explambda[k] <- exp(lambda[k])

}

for (j in 1:2){ # LOOP OVER POPULATIONS 1=adults; 2=children

d[j,1] <- 0 # treatment effect is zero for reference treatment

# pairwise RDs for all possible pair-wise comparisons

for (c in 1:(nt-1)){

for (k in (c+1):nt){

lnRR[j,c,k] <- d[j,k] - d[j,c]

RR[j,c,k] <- exp(d[j,k] - d[j,c])

}

}

# ranking on relative scale

for (k in 1:nt) {

# rk[j,k] <- nt+1-rank(d[j, ],k) # assumes events are "good"

rk[j,k] <- rank(d[j, ],k) # assumes events are "bad"

best[j,k] <- equals(rk[j,k],1) #calculate probability that treat k is best

# calculates probability that treat k is h-th best

for (h in 1:nt){ prob[j,h,k] <- equals(rk[j,k],h) }

}

} # close loop over populations

} # *** PROGRAM ENDS

*Suggested data format and initial values are the same as for the splitting model.*

Meta-regression model:

Fixed effects model – (Relative Risk)

# Binomial likelihood, log-Relative Risk

# Fixed effects model - 2 populations, meta-regression

model{ # *** PROGRAM STARTS

for(i in 1:ns){ # LOOP THROUGH STUDIES

mu[i] <- log(p[i,1])

p[i,1] ~ dunif(0,1) # vague priors for all trial baselines

for (k in 1:na[i]){ # LOOP THROUGH ARMS

r[i,k] ~ dbin(p[i,k],n[i,k]) # binomial likelihood

rhat[i,k] <- p[i,k] * n[i,k] # expected value of the numerators

# Deviance contribution

dev[i,k] <- 2 * (r[i,k] * (log(r[i,k])-log(rhat[i,k]))

+ (n[i,k]-r[i,k]) * (log(n[i,k]-r[i,k]) - log(n[i,k]-rhat[i,k])))

}

# model for linear predictor

for (k in 2:na[i]){

log(p[i,k]) <- mu[i] + min(delta[i,k], -log(p[i,1]))

delta[i,k] <- d[t[i,k]] - d[t[i,1]] + (beta[t[i,k]]-beta[t[i,1]]) * (adult[i]-mx) # mx set to zero for subgroups

}

# summed residual deviance contribution for this trial

resdev[i] <- sum(dev[i,1:na[i]])

}

totresdev <- sum(resdev[]) # Total Residual Deviance

#

d[1]<-0 # treatment effect is zero for reference treatment

**beta[1]** <- 0 # covariate effect is zero for reference treatment

# vague priors for treatment effects

for (k in 2:nt){

d[k] ~ dnorm(0,.0001)

beta[k] <- B # common covariate effect

}

B ~ dnorm(0,.0001) # vague prior for covariate effect

# treatment effect when covariate = z[j]

for (k in 1:nt){ # LOOP THROUGH TREATMENTS

for (j in 1:nz) { dz[j,k] <- d[k] + (beta[k]-beta[1])*(z[j]-mx) }

}

# pairwise RRs for all possible pair-wise comparisons

for (c in 1:(nt-1)){

for (k in (c+1):nt){

lnRR[c,k] <- d[k] - d[c]

RR[c,k] <- exp(d[k] - d[c])

# at covariate=z[j]

for (j in 1:nz) {

RRz[j,c,k] <- exp(dz[j,k] - dz[j,c])

lnRRz[j,c,k] <- (dz[j,k]-dz[j,c])

}

}

}

# ranking on relative scale

for (k in 1:nt) {

# rk[k] <- nt+1-rank(d[],k) # assumes events are "good"

rk[k] <- rank(d[],k) # assumes events are "bad"

best[k] <- equals(rk[k],1) #calculate probability that treat k is best

# calculates probability that treat k is h-th best

for (h in 1:nt){ prob[h,k] <- equals(rk[k],h) }

}

}

# *** PROGRAM ENDS

Random effects model (Relative risk)

# Binomial likelihood, log-Relative Risk

# Random effects model for multi-arm trials - 2 populations, meta-regression

model{ # *** PROGRAM STARTS

for(i in 1:ns){ # LOOP THROUGH STUDIES

w[i,1] <- 0 # adjustment for multi-arm trials is zero for control arm

delta[i,1] <- 0 # treatment effect is zero for control arm

**mu[i] <- log(p[i,1])**

**p[i,1] ~ dunif(0,1)** # vague priors for all trial baselines

for (k in 1:na[i]) { # LOOP THROUGH ARMS

r[i,k] ~ dbin(p[i,k],n[i,k]) # binomial likelihood

rhat[i,k] <- p[i,k] * n[i,k] # expected value of the numerators

# Deviance contribution

dev[i,k] <- 2 * (r[i,k] * (log(r[i,k])-log(rhat[i,k]))

+ (n[i,k]-r[i,k]) * (log(n[i,k]-r[i,k]) - log(n[i,k]-rhat[i,k])))

}

# summed residual deviance contribution for this trial

resdev[i] <- sum(dev[i,1:na[i]])

for (k in 2:na[i]) { # LOOP THROUGH ARMS

**log(p[i,k]) <- mu[i] + min(delta[i,k], -log(p[i,1]))**

# trial-specific LOR distributions

delta[i,k] ~ dnorm(md[i,k],taud[i,k])

# mean of LOR distributions (with multi-arm trial correction)

md[i,k] <- d[t[i,k]] - d[t[i,1]] + sw[i,k] + (beta[t[i,k]]-beta[t[i,1]]) * (adult[i]-mx) # mx set to zero

# precision of LOR distributions (with multi-arm trial correction)

taud[i,k] <- tau *2*(k-1)/k

# adjustment for multi-arm RCTs

w[i,k] <- (delta[i,k] - d[t[i,k]] + d[t[i,1]])

# cumulative adjustment for multi-arm trials

sw[i,k] <- sum(w[i,1:k-1])/(k-1)

}

}

totresdev <- sum(resdev[]) # Total Residual Deviance

d[1]<-0 # treatment effect is zero for reference treatment

**beta[1]** <- 0 # covariate effect is zero for reference treatment

# vague priors for treatment effects

for (k in 2:nt){

d[k] ~ dnorm(0,.0001)

beta[k] <- B # common covariate effect

}

sd ~ dunif(0,2) # vague prior for between-trial SD

tau <- pow(sd,-2) # between-trial precision = (1/between-trial variance

B ~ dnorm(0,.0001) # vague prior for covariate effect

# treatment effect when covariate = z[j]

for (k in 1:nt){ # LOOP THROUGH TREATMENTS

for (j in 1:nz) { dz[j,k] <- d[k] + (beta[k]-beta[1])*(z[j]-mx) }

}

# pairwise RRs for all possible pair-wise comparisons

for (c in 1:(nt-1)){

for (k in (c+1):nt){

lnRR[c,k] <- d[k] - d[c]

RR[c,k] <- exp(d[k] - d[c])

# at covariate=z[j]

for (j in 1:nz) {

RRz[j,c,k] <- exp(dz[j,k] - dz[j,c])

lnRRz[j,c,k] <- (dz[j,k]-dz[j,c])

}

}

}

# ranking on relative scale

for (k in 1:nt) {

# rk[k] <- nt+1-rank(d[],k) # assumes events are "good"

rk[k] <- rank(d[],k) # assumes events are "bad"

best[k] <- equals(rk[k],1) #calculate probability that treat k is best

# calculates probability that treat k is h-th best

for (h in 1:nt){ prob[h,k] <- equals(rk[k],h) }

}

} # *** PROGRAM ENDS

Example of data structure:

list(ns=8, nt=2 , z=c(1), nz=1, mx=0 ) # indicating 8 studies comparing two treatments, one covariate, and mx set to 0. Adults are coded as 0 in the data table and children as 1.

t[,1] r[,1] n[,1] t[,2] r[,2] n[,2] adult[] na[] #study

1 88 167 2 62 173 0 2 # 1

1 93 212 2 68 209 0 2 # 2

1 189 418 2 138 430 0 2 # 3

1 165 498 2 115 502 0 2 # 4

1 40 44 2 41 52 1 2 # 5

1 17 18 2 20 28 1 2 # 6

1 122 152 2 94 155 1 2 # 7

1 48 82 2 25 82 1 2 # 8

END

Initial values

**#FE chains**

#chain 1

list(d=c(NA,0), B=0 ) # add beta into initial values

#chain 2

list(d=c(NA,1), B=-1)

#chain 3

list(d=c(NA,2), B=0)

**#RE chains**

#for RE model add in sd.

#chain 1

list(d=c(NA,0), sd=0.5,B=0) # add beta into initial values

#chain 2

list(d=c(NA,1),sd=0.1 , B=-1)

list(d=c(NA,2),sd=0.1 , B=0)

Meta-regression model with the inclusion of a baseline risk covariate:

Here baseline risk acts as a proxy for underlying or unmeasured patient-level covariates so the approach so you are able to infer whether the average baseline risk for a given trial is associated with treatment effect.

Fixed effects model (Relative risk)

# Binomial likelihood, log-Relative Risk

# Fixed effects model - 2 populations, meta-regression

model{ # *** PROGRAM STARTS

for(i in 1:ns){ # LOOP THROUGH STUDIES

mu[i] <- log(p[i,1])

p[i,1] ~ dunif(0,1) # vague priors for all trial baselines

for (k in 1:na[i]){ # LOOP THROUGH ARMS

r[i,k] ~ dbin(p[i,k],n[i,k]) # binomial likelihood

rhat[i,k] <- p[i,k] * n[i,k] # expected value of the numerators

# Deviance contribution

dev[i,k] <- 2 * (r[i,k] * (log(r[i,k])-log(rhat[i,k]))

+ (n[i,k]-r[i,k]) * (log(n[i,k]-r[i,k]) - log(n[i,k]-rhat[i,k])))

}

# model for linear predictor

for (k in 2:na[i]){

log(p[i,k]) <- mu[i] + min(delta[i,k], -log(p[i,1]))

delta[i,k] <- d[t[i,k]] - d[t[i,1]] + (beta[t[i,k]]-beta[t[i,1]]) * (mu[i]-mx)

# mx set to =-LOG(0.556)/1-0.556

}

# summed residual deviance contribution for this trial

resdev[i] <- sum(dev[i,1:na[i]])

}

totresdev <- sum(resdev[]) # Total Residual Deviance

#

d[1]<-0 # treatment effect is zero for reference treatment

**beta[1]** <- 0 # covariate effect is zero for reference treatment

# vague priors for treatment effects

for (k in 2:nt){

d[k] ~ dnorm(0,.0001)

beta[k] <- B # common covariate effect

}

B ~ dnorm(0,.0001) # vague prior for covariate effect

# treatment effect when covariate = z[j]

for (k in 1:nt){ # LOOP THROUGH TREATMENTS

for (j in 1:nz) { dz[j,k] <- d[k] + (beta[k]-beta[1])*(z[j]-mx) }

}

# pairwise RRs for all possible pair-wise comparisons

for (c in 1:(nt-1)){

for (k in (c+1):nt){

lnRR[c,k] <- d[k] - d[c]

RR[c,k] <- exp(d[k] - d[c])

# at covariate=z[j]

for (j in 1:nz) {

RRz[j,c,k] <- exp(dz[j,k] - dz[j,c])

lnRRz[j,c,k] <- (dz[j,k]-dz[j,c])

}

}

}

# ranking on relative scale

for (k in 1:nt) {

# rk[k] <- nt+1-rank(d[],k) # assumes events are "good"

rk[k] <- rank(d[],k) # assumes events are "bad"

best[k] <- equals(rk[k],1) #calculate probability that treat k is best

# calculates probability that treat k is h-th best

for (h in 1:nt){ prob[h,k] <- equals(rk[k],h) }

}

}

# *** PROGRAM ENDS

Random effects model (Relative risk)

# Binomial likelihood, log-Relative Risk

# Random effects model for multi-arm trials - 2 populations, meta-regression

model{ # *** PROGRAM STARTS

for(i in 1:ns){ # LOOP THROUGH STUDIES

w[i,1] <- 0 # adjustment for multi-arm trials is zero for control arm

delta[i,1] <- 0 # treatment effect is zero for control arm

**mu[i] <- log(p[i,1])**

**p[i,1] ~ dunif(0,1)** # vague priors for all trial baselines

for (k in 1:na[i]) { # LOOP THROUGH ARMS

r[i,k] ~ dbin(p[i,k],n[i,k]) # binomial likelihood

rhat[i,k] <- p[i,k] * n[i,k] # expected value of the numerators

# Deviance contribution

dev[i,k] <- 2 * (r[i,k] * (log(r[i,k])-log(rhat[i,k]))

+ (n[i,k]-r[i,k]) * (log(n[i,k]-r[i,k]) - log(n[i,k]-rhat[i,k])))

}

# summed residual deviance contribution for this trial

resdev[i] <- sum(dev[i,1:na[i]])

for (k in 2:na[i]) { # LOOP THROUGH ARMS

**log(p[i,k]) <- mu[i] + min(delta[i,k], -log(p[i,1]))**

# trial-specific LOR distributions

delta[i,k] ~ dnorm(md[i,k],taud[i,k])

# mean of LOR distributions (with multi-arm trial correction)

md[i,k] <- d[t[i,k]] - d[t[i,1]] + sw[i,k] + (beta[t[i,k]]-beta[t[i,1]]) * (adult[i]-mx) # mx set to zero

# precision of LOR distributions (with multi-arm trial correction)

taud[i,k] <- tau *2*(k-1)/k

# adjustment for multi-arm RCTs

w[i,k] <- (delta[i,k] - d[t[i,k]] + d[t[i,1]])

# cumulative adjustment for multi-arm trials

sw[i,k] <- sum(w[i,1:k-1])/(k-1)

}

}

totresdev <- sum(resdev[]) # Total Residual Deviance

d[1]<-0 # treatment effect is zero for reference treatment

**beta[1]** <- 0 # covariate effect is zero for reference treatment

# vague priors for treatment effects

for (k in 2:nt){

d[k] ~ dnorm(0,.0001)

beta[k] <- B # common covariate effect

}

sd ~ dunif(0,2) # vague prior for between-trial SD

tau <- pow(sd,-2) # between-trial precision = (1/between-trial variance

B ~ dnorm(0,.0001) # vague prior for covariate effect

# treatment effect when covariate = z[j]

for (k in 1:nt){ # LOOP THROUGH TREATMENTS

for (j in 1:nz) { dz[j,k] <- d[k] + (beta[k]-beta[1])*(z[j]-mx) }

}

# pairwise RRs for all possible pair-wise comparisons

for (c in 1:(nt-1)){

for (k in (c+1):nt){

lnRR[c,k] <- d[k] - d[c]

RR[c,k] <- exp(d[k] - d[c])

# at covariate=z[j]

for (j in 1:nz) {

RRz[j,c,k] <- exp(dz[j,k] - dz[j,c])

lnRRz[j,c,k] <- (dz[j,k]-dz[j,c])

}

}

}

# ranking on relative scale

for (k in 1:nt) {

# rk[k] <- nt+1-rank(d[],k) # assumes events are "good"

rk[k] <- rank(d[],k) # assumes events are "bad"

best[k] <- equals(rk[k],1) #calculate probability that treat k is best

# calculates probability that treat k is h-th best

for (h in 1:nt){ prob[h,k] <- equals(rk[k],h) }

}

} # *** PROGRAM ENDS

Example of how to input data:

list(ns=8, nt=2 , z=c(1), nz=1, mx=-0.301 ) # indicating 8 studies comparing two treatments, one covariate, and mx set to 0.301. Adults are coded as 0 in the data table and children as 1.

t[,1] r[,1] n[,1] t[,2] r[,2] n[,2] adult[] na[] #study

1 88 167 2 62 173 0 2 # 1

1 93 212 2 68 209 0 2 # 2

1 189 418 2 138 430 0 2 # 3

1 165 498 2 115 502 0 2 # 4

1 40 44 2 41 52 1 2 # 5

1 17 18 2 20 28 1 2 # 6

1 122 152 2 94 155 1 2 # 7

1 48 82 2 25 82 1 2 # 8

END

Initial values

**#FE chains**

#chain 1

list(d=c(NA,0), B=0 ) # add beta into initial values

#chain 2

list(d=c(NA,1), B=-1)

#chain 3

list(d=c(NA,2), B=0)

**#RE chains**

#for RE model add in sd.

#chain 1

list(d=c(NA,0), sd=0.5,B=0) # add beta into initial values

#chain 2

list(d=c(NA,1),sd=0.1 , B=-1)

list(d=c(NA,2),sd=0.1 , B=0)
